# P-1. Impact of Management Bundles on the Prognosis of Candidemia Patients: A Multicenter Retrospective Cohort Study (2016–2023)

**DOI:** 10.1093/ofid/ofaf695.232

**Published:** 2026-01-11

**Authors:** Hidemasa Akazawa, Shinnosuke Fukushima, Hideharu Hagiya

**Affiliations:** Okayama University Hospital, Okayama City, Okayama, Japan; Department of Bacteriology, Okayama University Graduate School of Medicine, Dentistry and Pharmaceutical Sciences, Okayama, Okayama, Japan; Okayama University Hospital, Okayama City, Okayama, Japan

## Abstract

**Background:**

Candidemia is a severe systemic infection with a high mortality risk, which has been designated as a critical global health concern by the World Health Organization. To validate the clinical efficacy of a standardized Candida care bundle implementation, we conducted a multicenter observational study.

**Methods:**

In this retrospective multicenter cohort study, 230 patients with candidemia across nine Japanese hospitals (2016–2023) were evaluated. Compliance was assessed based on five components: central venous catheter removal within 24 h of diagnosis, appropriate initial antifungal therapy, ophthalmologic examination, follow-up blood cultures until clearance, and antifungal therapy for at least two weeks post-clearance. Patients were stratified into high (4–5 points) and low (0–3 points) bundle compliance groups. The primary outcome was set as 30-day mortality, which were analyzed by Kaplan–Meier curves and the log-rank test. To identify prognostic factors, a multivariate Cox proportional hazards model was applied by incorporating potential variables.

**Results:**

The overall mortality rate was 23.5%. Mortality occurred in 14 of 160 patients (8.8%) in the high-compliance group and 40 of 70 (57.1%) in the low-compliance group. High compliance was associated with a significantly lower risk of 30-day mortality (hazard ratio [HR], 0.09; 95% confidence interval [CI], 0.05–0.17; p < 0.001). Central line-associated blood stream infection was identified as a significant predictor of mortality (HR, 2.25; 95% CI, 1.21–4.20; p = 0.01), whereas age and sex were not associated with prognosis (HR, 1.04; 95% CI, 0.60–1.79; p = 0.89 and HR, 1.25; 95% CI, 0.77–2.42; p = 0.29, respectively).

**Conclusion:**

High bundle compliance significantly improved survival in candidemia. These findings underscore the importance of adherence to evidence-based management.

**Disclosures:**

All Authors: No reported disclosures

